# Influence of meniscus on cartilage and subchondral bone features of knees from older individuals: A cadaver study

**DOI:** 10.1371/journal.pone.0181956

**Published:** 2017-08-10

**Authors:** Sébastien Touraine, Hamid Bouhadoun, Klaus Engelke, Jean Denis Laredo, Christine Chappard

**Affiliations:** 1 B2OA, UMR CNRS 7052, University Paris Diderot, Paris, France; 2 Service de Radiologie Ostéo-Articulaire, Hôpital Lariboisière, Paris, France; 3 Institute of Medical Physics, University of Erlangen-Nürnberg, Erlangen, Germany; Université de Poitiers, FRANCE

## Abstract

**Objective:**

Cartilage and subchondral bone form a functional unit. Here, we aimed to examine the effect of meniscus coverage on the characteristics of this unit in knees of older individuals.

**Methods:**

We assessed the hyaline cartilage, subchondral cortical plate (SCP), and subchondral trabecular bone in areas covered or uncovered by the meniscus from normal cadaver knees (without degeneration). Bone cores harvested from the medial tibial plateau at locations uncovered (central), partially covered (posterior), and completely covered (peripheral) by the meniscus were imaged by micro-CT. The following were measured on images: cartilage volume (Cart.Vol, mm^3^) and thickness (Cart.Th, mm); SCP thickness (SCP.Th, μm) and porosity (SCP.Por, %); bone volume to total volume fraction (BV/TV, %); trabecular thickness (Tb.Th, μm), spacing (Tb.Sp, μm), and number (Tb.N, 1/mm); structure model index (SMI); trabecular pattern factor (Tb.Pf); and degree of anisotropy (DA).

**Results:**

Among the 28 specimens studied (18 females) from individuals with mean age 82.8±10.2 years, cartilage and SCP were thicker at the central site uncovered by the meniscus than the posterior and peripheral sites, and Cart.Vol was greater. SCP.Por was highest in posterior samples. In the upper 1–5 mm of subchondral bone, central samples were characterized by higher values for BV/TV, Tb.N, Tb.Th, and connectivity (Tb.Pf), a more plate-like trabecular structure and lower anisotropy than with other samples. Deeper down, at 6–10 mm, the differences were slightly higher for Tb.Th centrally, DA peripherally and SMI posteriorly.

**Conclusions:**

The coverage or not by meniscus in the knee of older individuals is significantly associated with Cart.Th, SCP.Th, SCP.Por and trabecular microarchitectural parameters in the most superficial 5 mm and to a lesser extent the deepest area of subchondral trabecular bone. These results suggest an effect of differences in local loading conditions. In subchondral bone uncovered by the meniscus, the trabecular architecture resembles that of highly loaded areas.

## Introduction

Knee menisci play an important role in load bearing, shock absorption, and joint congruity and stability [1]. Their role in attenuating and distributing impact loads may be greater than that of the hyaline cartilage [2]. According to a study of human cadaver knees, 58% of the load is transmitted through the meniscus and 42% through the uncovered cartilage [3]. Contact pressures below the meniscus increased by 23% after partial meniscectomy in a knee simulator [4] and peak contact pressure by 25% with a posterior root tear in the medial meniscus of human cadaver knees [5]. More generally, meniscal damage is strongly associated with higher bone mineral density (BMD) in the tibial plateau, as measured by CT, in keeping with a response to increased loading [6,7]. Meniscectomy, whether partial or total, is associated with knee osteoarthritis progression [8]. Similarly, posterior root tears in the medial meniscus often lead to meniscal extrusion, which is a risk factor for osteoarthritis progression [9,10].

The 3D microarchitecture of human subchondral bone has been studied in normal and osteoarthritic hips [11–13] and knees [14–16] by micro-CT. Depth-dependent microstructure differences in the uppermost 6 mm of trabecular bone have been described between the medial and lateral compartments of normal and osteoarthritic knees, with differences disappearing below 6 mm [15]. Subchondral bone architecture would be expected to differ between areas covered versus uncovered by meniscus because meniscus has an impact on mechanical loading distribution. Such differences were identified in an experimental study of porcine tibial plateaus, which also documented variations in cartilage thickness and biomechanical characteristics of both cartilage and trabecular bone [17]. However, to our knowledge, these findings have not been confirmed in humans. Furthermore, no studies of humans have compared areas covered and uncovered by meniscus by evaluating cartilage thickness, subchondral cortical plate (SCP) and subchondral trabecular bone (STB) microarchitecture as well as differences in 3D subchondral bone architecture.

Here, we looked for associations between coverage by the meniscus and characteristics of the medial compartment bone-cartilage interface in cadaver knees from older individuals with little or no evidence of osteoarthritis. Hyaline cartilage thickness, SCP thickness, and STB microarchitectural variables in the medial tibial plateau were measured in volumes of interest (VOIs). We focused on the medial compartment, which carries 75% of the load across the knee [18] and is most affected by osteoarthritis [19] and in which bone changes related to osteoarthritis are severe [15].

## Materials and methods

### Ethics statement

The study was approved by our institutional review board and conducted according to pertinent protocols established by the Human Ethics Committee of the Institut d’Anatomie, unité de formation et de recherche biomédicale des Saints Pères, Université Paris Descartes. The subjects willed their body to science and were anonymous.

### Specimen preparation and micro-CT imaging

We studied 40 left human cadaver knees obtained from the Institute of Anatomy, Paris, France. All 40 donors were white, The knees were selected on the basis of posterior-anterior radiographs (Axiom Luminos, Siemens, Munich, Germany) scored by the Kellgren-Lawrence system [20]. Finally, we obtained 29 normal knee specimens with scores 0 (normal) or 1 (doubtful degenerative changes; 18 individuals were females with mean age 85.7±7.5 years and 11 were males with mean age 77.9±12.0 years. No information was available on cause of death, previous illnesses, walking ability, or medication exposures.

After soft-tissue removal, knees were stored at -20°C. The knees were dissected and a macroscopic grading system was used to characterize degeneration of the menisci before their removal [21]. One of the 29 knee specimens was excluded because it presented clear cartilage and meniscus impairments. The final sample consisted of 28 knee specimens (18 females). The tibial plateaus were excised parallel to the joint surface at a depth of 3 cm.

**Fig 1:**
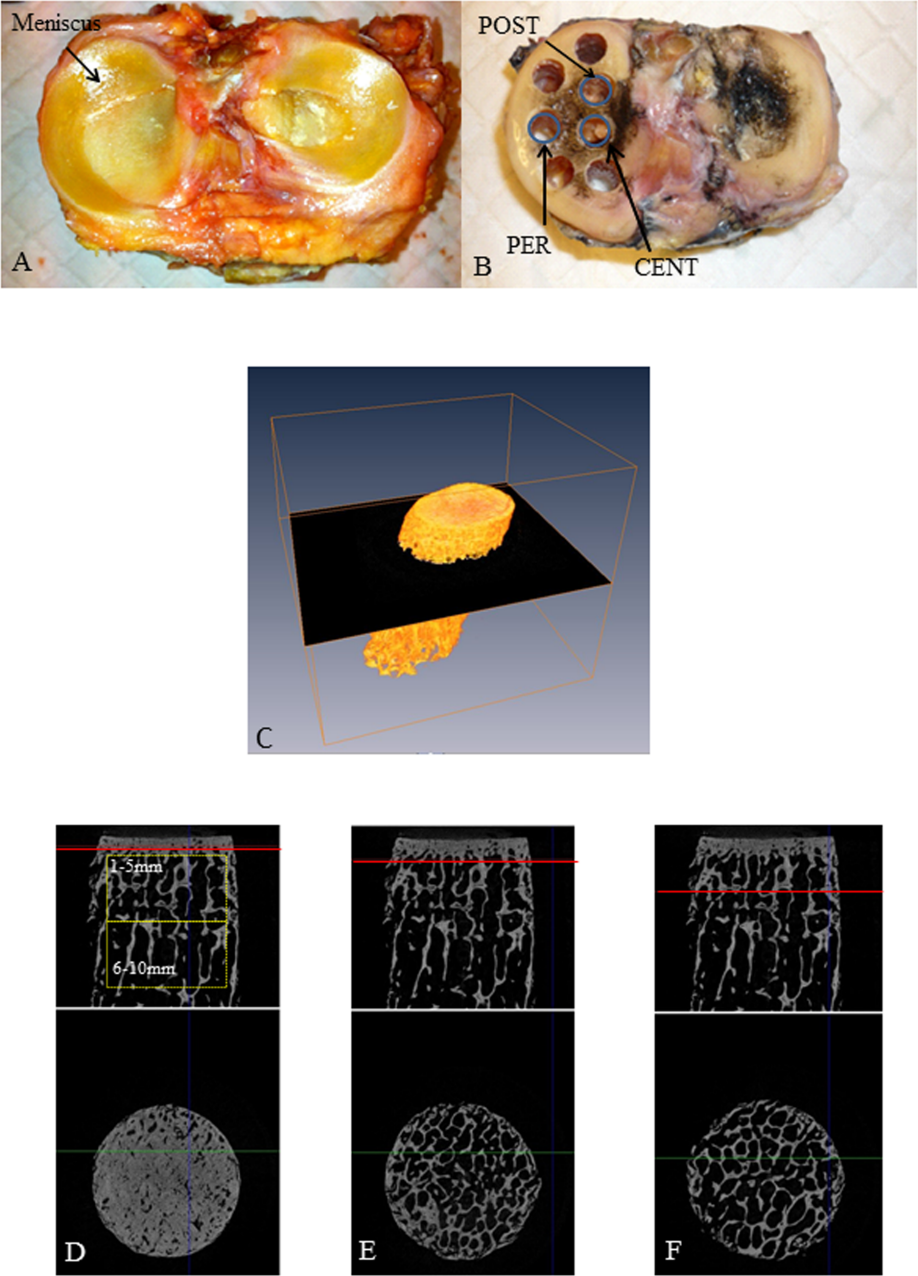
Sampling sites and micro-CT analyses. (A) Tibial plateau with meniscus. (B) Tibial plateau without meniscus and osteochondral plug sampling sites. (C) Post-acquisition rotation of the micro-CT images ensured that each slice was perpendicular to the cartilage surface. (D-E-F) Structure of the subchondral plate (SCP) and subchondral trabecular bone (STB) and volumes of interest (VOIs, in boxes) used to analyze the subchondral components; transverse slices through the SCP, at the interface between the SCP and STB, and within the STB. Red line: the slice located below.

To improve cartilage visualization, notably the detection of cartilage fibrillation, the joint surfaces were stained with waterproof black India ink (Sanford Rotring GmbH, Hamburg, Germany) [22], and cartilage degradation was assessed by the modified Outerbridge classification [23] (Fig 1A). The tibial plateau measurements were measured in millimeters by using a precision caliper (OTMT, New York, USA).

Three bone plugs were removed from the medial tibial plateau by using a trephine of 7 mm in internal diameter under constant water irrigation (BROT, Argenteuil, France). One plug was taken from the periphery (PER) and another from the central area (CENT), both midway between the anterior and posterior edges of the medial tibial plateau. The third plug was taken from a site posterior to the central sampling site (POST), at a mean of 10.3 ±2 mm from the posterior border (Fig 1B). PER represents complete coverage, POST partial coverage and CENT no coverage by the meniscus.

Each plug was scanned twice (SkyScan 1172, Bruker, Billerica, MA) to independently optimize the contrast of cartilage and bone. 3D datasets were reconstructed, with voxel size 10.2 μm and 0–255 gray-level range. For the cartilage scan, each plug was placed in a plastic tube that was immersed in a mineral-oil bath (Sigma-Aldrich, St Louis, MO). The micro-CT datasets were acquired at 37 kV and 100 μA as described in detail elsewhere [24]. For the bone scan, bone plugs were completely defatted before acquisition by using supercritical CO_2_ [25], at 80 kV and 100 μA, with a 1-mm–thick aluminum filter.

### Cartilage, SCP and STB analyses

For cartilage volume (Cart.Vol, mm^3^) measurements, the cartilage surface was contoured manually every 25–50 slices. Contours were interpolated in the interleaving slices (SkyScan, CtAn). Cartilage thickness (Cart.Th, mm) was measured by the sphere method (SkyScan, CtAn). Briefly, for each point of the segmented cartilage, the diameter of the largest sphere that fit completely inside the object was measured [26].

The tibial plateau does not usually form a right angle with the tibial axis; instead, the angle varies among sites on the plateau. Therefore, we rotated the micro-CT datasets (AMIRA software, FEI, OR) to ensure that, in the 1×1mm analysis (described below), each slice was parallel to the cartilage surface (Fig 1C).

SCP thickness (SCP.Th, μm) was measured by the same technique as for the cartilage analysis (i.e., manual segmentation combined with the sphere method). Porosity (SCP.Por, %) was determined after binarization (with a threshold of 60).

STB microarchitecture was analyzed in two cylindrical VOIs, each 6 mm in diameter, one extending from 1–5 mm beneath the SCP (STB_1–5 mm_) and the other 6–10 mm beneath the SCP (STB_6–10 mm_) (Fig 1D). A 3D analysis was also performed 1×1 mm from the subchondral plate to a depth of 10 mm. Standard and advanced morphological parameters were assessed in 3D binarized volumes (with a threshold of 60). Relative bone volume (BV/TV, %) and surface density (BS/TV, 1/mm) were measured.

Trabecular number (Tb.N, 1/mm) was calculated as the mean distance between the medial axes of the structure [27]. Trabecular thickness (Tb.Th, μm) and trabecular separation (Tb.Sp, μm) were derived by the model-independent method [26]. Other variables were the structure model index (SMI), the trabecular pattern factor (Tb.Pf), and the degree of anisotropy (DA). SMI quantifies plates versus rods: values are 0 for a perfect plate structure and 3 for a perfect rod structure [28]. Tb.Pf is an index of connectivity, with higher values indicating lower connectivity [29]. DA, based on the mean intercept method, defines the orientation of the trabecular structure: the value is 1 for isotropic structures and >1 for anisotropic structures [30].

### Statistical analyses

Data by gender were compared by non-paired Student *t* test or Mann-Whitney test for non-normally distributed variables. Between-site differences in cartilage, SCP, and STB structure were assessed by one-way ANOVA for normally distributed variables and Kruskal-Wallis test otherwise, followed by Bonferroni correction for multiple comparisons. The same tests were used to compare the results of the 1×1mm analysis. Values measured at different depths from the subchondral plate (1–5 vs 6–10 mm) were compared by paired Student *t* test for normally distributed data and Wilcoxon test otherwise. Correlations between cartilage thickness and SCP or STB characteristics were assessed by Pearson correlation coefficient for normally distributed variables and Spearman coefficient for non-normally distributed variables. P<0.05 was considered statistically significant. All statistical analyses were performed with NCSS software (NCSS, Kaysville, UT).

## Results

### Meniscus, cartilage, and SCP

For the 28 specimens studied (18 females), Table 1 reports the features of the meniscus, cartilage and bone at the PER, POST, and CENT sites. When all three sites and both genders were pooled, mean cartilage thickness was 2.0±0.42 mm (range 1.28–3.09 mm). Cart.Vol and Cart.Th were greater in the CENT than PER and POST sites. Cart.Th was greater at the CENT than PER site for 96.4% of knees and greater than at the POST site for 82.1%. SCP.Th was greater at the CENT site than the other two sites. SCP.Por was highest at the POST site.

**Table 1:** Meniscus, cartilage classifications and micro-CT results for cartilage and subchondral plate in all knees (n = 28) by sample site.

		Sample site				
		PER	CENT	POST	P value^a^	Bonferroni test^b^
**Meniscus degeneration grades**						
Grade 1 Grade 2 Grade 3	n = 23; n = 4 for calcification n = 2 n = 3					
**Modified Outerbridge classification (23)**						
Grade 0		n = 18	n = 8	n = 18		
Grade 1		n = 10	n = 15	n = 10		
Grade 2		n = 0	n = 5	n = 0		
**Tibial plateau measurements**						
Length, mm	85.5±23.2 (55–75)					
Width, mm	64.9±4.9 (33–54)					
**Micro-CT results**						
***Cartilage***						
Volume (mm^3^)		68.2±11.4 (37.0±88.4)	104.6±24.1 (62.0–166.7)	81.5±25.0 (20.3–127.0)	10^−6^	CENT vs. PER/POST
Thickness, μm		1.8±0.2 (1.3–2.4)	2.3±0.4 (1.8–3.1)	1.9±0.4 (0.9–2.8)	10^−6^	CENT vs. PER/POST
***Subchondral plate***						
Thickness, μm		456±165 (225–788)	670±194 (348–1096)	438±195 (137–996)	2·10^−6^	CENT vs. PER/POST
Porosity, %		17.9±7.8 (5.4–35.5)	18.2±4.6 (10.5–27.7)	24.5±12.0 (10.5–74.0)	0.004	POST vs. CENT/PER

Data are mean±SD (range) values of cartilage and subchondral bone plate variables at three sampling sites in the medial tibial plateau: central (CENT, uncovered), peripheral (PER, fully covered), and posterior (POST, partially covered).

^a^Normally distributed variables were compared by ANOVA and non-normally distributed variables by Kruskal-Wallis test.

^b^The site named first differed significantly from the other two sites pooled.

Table 2 describes the cartilage and subchondral plate variables measured at the three sites for both genders. Cart.Th was greater for males than females at the POST and PER sites (P = 0.01 and P = 0.002, respectively).

**Table 2:** Micro-CT results for cartilage and subchondral plate (SCP) at the three sample sites by gender.

	Sample site					
	POST		CENT		PER	
	*Male*	*Female*	*Male*	*Female*	*Male*	*Female*
***Cartilage***						
Volume (mm^3^)	94.1±21.6	79.9±21.9*	104±26.3	105±24.4	72.5±11.2	66.3±10.2*
Thickness, μm	2.1±0.4	1.8±0.3**	2.4±0.4	2.3±0.4	2.0±0.3	1.7±0.2**
***Subchondral plate***						
Thickness, μm	0.46±0.15	0.39±0.17	0.72±0.23	0.62±0.15	0.48±0.18	0.44±0.15
Porosity, %	23.3±5.8	25.9±14.4	19.1±4.3	17.6±4.9	18.1±7.8	17.9±8.0

Data are mean±SD.

* P<0.05

** P< 0.01 compared to males.

### Subchondral trabecular bone

Fig 1D, 1E and 1F illustrates the visible change in STB microarchitecture at 5 mm. Table 3 describes the STB architectural variables measured at the three sites and both depths (VOIs 1–5 and 6–10 mm below the SCP) for both genders.

**Table 3:** Subchondral trabecular bone (STB) architectural variables by sample site and gender and depth (1–5 and 6–10 mm below the SCP).

	Sample site					
	POST		CENT		PER	
VOI/variable	*Male*	*Female*	*Male*	*Female*	*Male*	*Female*
***1–5 mm***						
BV/TV (%)	28.3±8.3	20.2±7.4ǂ	35.7±8.9	26.0±7.8**	24.7±5.6	*19*.*3±7*.4*
BS/TV (1/mm)	5.3±0.7	4.8±2.8	6.1±0.7	5.6±1.0	5.5±1.0	4.8±0.0*
Tb.Th (μm)	183±31	145±24**	198±42	158±22**	155±21	137±23*
Tb.N (1/mm)	1.5±0.2	1.4±0.3	1.8±0.2	1.6±0.3	1.6±0.3	1.4±0.3
Tb.Sp (μm)	492±57	532±93	448±59	486±87	487±66	539±87
SMI	1.1±0.6	1.2±0.4	0.3±0.6	*0*.*7±0*.9**	0.8±0.5	1.0±0.6
Tb.Pf	2.6±2.8	4.1±2.8*	-1.3±1.7	*1*.*0±3*.5**	1.3±2.2	3.5±2.3*
DA	1.4±0.1	*1*.7±0.3*	1.5±0.2	1.4±0.2	1.7±0.2	1.8±0.3
***6–10 mm***						
BV/TV (%)	16.7±5.4	13.4±4.1*	*21*.*3±5*.*7*	14.2±5.0**	17.1±4.5	*12*.*6±5*.3*
BS/TV (1/mm)	*4*.*0±0*.*9*	3.7±0.7	4.1±0.3	3.6±0.7**	4.3±0.8	3.5±0.7**
Tb.Th (μm)	148±26	128±18*	179±37	137±26**	137±19	123±27
Tb.N (1/mm)	*1*.*1±0*.*2*	*1*.*0±0*.*2*	1.2±0.1	1.0±0.2**	1.2±0.2	*1*.*0±0*.2*
Tb.Sp (μm)	*625±75*	648±88	644±38	677±108	591±70	656±108
SMI	*1*.*7±0*.*3*	1.5±0.2	1.1±0.5	1.4±0.2*	1.2±0.4	1.3±0.4
Tb.Pf	5.7±1.7	5.7±2.1	2.4±1.6	5.1±1.9ǂ	3.9±2.0	*5*.*5±3*.*1*
DA	1.8±0.5	2.1±0.5	1.9±0.4	2.1±0.4	*2*.*5±0*.*4*	*2*.*4±0*.*7*

Data are mean±SD of morphological parameters in the two volumes of interest (VOI): 1–5 and 6–10 mm below the SCP.

* P<0.05

** P< 0.01

**ǂ***P*<10^−4^ compared to males.

Normally distributed variables were compared by non-paired Student *t* test and non-normally distributed variables (in italics) by Mann Whitney test.

POST, posterior sampling site (partially covered); CENT, central sampling site (uncovered); PER, peripheral sampling site (fully covered).

At the 1–5 mm depth, BV/TV and Tb.Th were significantly greater for males than females at all sites (P = 0.01 to 0.003 and 0.02 to 0.001, respectively). Tb.Pf was greater for males than females at all sites (P = 0.03) as was BS/TV at the PER site (P = 0.04). SMI values were lower for males at the CENT site and DA values were lower at POST site.

At the 6–10 mm depth, BV/TV was significantly greater for males than females at all sites (P = 0.04 to 0.001), Tb.Th was greater at POST and CENT sites (P = 0.01 and P = 0.001, respectively) and BS/TV was greater for males than females at the CENT and PER sites (P = 0.01). Tb.N was greater for males than females at the CENT and PER sites (P = 0.02 and P = 0.005, respectively). SMI and Tb.Pf values were lower for males than females at the CENT site (P = 0.002 and P = 0.001, respectively).

Table 4 describes the STB architectural variables measured at the three sites and at both depths (1–5 and 6–10 mm below the SCP). At 1–5 mm, BV/TV, BS/TV, Tb.N were significantly greater at the CENT than PER and POST sites (P≤0.009). Tb.Sp did not differ among the three sites, but Tb.Th was significantly greater at the CENT than PER site (P = 0.005), and Tb.Pf value was significantly lower at the CENT than PER and POST sites (P = 0.0003). DA was significantly greater at the PER than CENT site (P = 0.0008).

**Table 4:** Micro-architectural changes of the STB by sample site and depth for all knees (n = 28).

	Sample site				
VOI/variable	POST	CENT	PER	P value^a^	Multiple comparison tests^b^
***1–5 mm***					
BV/TV (%)	23.1±8.5ǂ	29.5±9.3ǂ	21.2±7.2ǂ	*0*.*001*	CENT vs PER/POST
BS/TV (1/mm)	5.0±1.0ǂ	5.8±0.9ǂ	5.1±1.0ǂ	*0*.*007*	CENT vs PER/POST
Tb.Th (μm)	158±32ǂ	172±36ǂ	143±24**	*0*.*004*	CENT vs PER
Tb.N (1/mm)	1.4±0.3ǂ	1.7±0.3ǂ	1.4±0.3ǂ	*0*.*003*	CENT vs PER/POST
Tb.Sp (μm)	518±83ǂ	472±78ǂ	520±82ǂ	*ns*	
SMI	1.2±0.4ǂ	0.5±0.8ǂ	1.0±0.6ǂ	*0*.*001*	CENT vs POST
Tb.Pf	3.6±2.8ǂ	0.2±3.2ǂ	2.8±-3.1ǂ	*0*.*0003*	CENT vs POST/PER
DA	1.6±0.3ǂ	1.4±0.2ǂ	1.8±0.3ǂ	*0*.*001*	CENT vs PER
***6–10 mm***					
BV/TV (%)	14.6±4.8	16.8±6.2	14.2±5.5	*0*.*0005*	CENT vs PER/POST
BS/TV (1/mm)	3.8±0.7	3.8±0.6	3.8±0.8	ns	
Tb.Th (μm)	135±23	152±36	129±26	*0*.*01*	CENT vs PER
Tb.N (1/mm)	1.1±0.2	1.1±0.2	1.1±0.3	ns	
Tb.Sp (μm)	640±83	665±90	633±100	ns	
SMI	1.5±0.3	1.3±0.4	1.2±0.4	0.02	POST vs PER
Tb.Pf	5.7±1.9	4.1±2.2	4.9±2.8	ns	
DA	2.0±0.5	2.0±0.4	2.5±0.6	*0*.*002*	PER vs POST/CENT

Data are mean±SD for the two volumes of interest (VOIs): 1–5 mm and 6–10 mm below the SCP.

** P< 0.01

ǂ*P*<10^−4^ comparing the two depths.

^a^Normally distributed variables were compared by ANOVA and non-normally distributed variables by Kruskal-Wallis test (in italics).

^b^The site named first differed significantly from the other two sites pooled.

POST, posterior sampling site (partially covered); CENT, central sampling site (uncovered); PER, peripheral sampling site (fully covered).

At 6–10 mm, BV/TV, Tb.Th, SMI and DA differed significantly across sites: BV/TV was greater at the CENT site than other sites. Tb.Th was significantly greater at the CENT than PER site (P = 0.01), DA was greater at the PER site than other sites (P = 0.001) and SMI was greater at the POST site than PER site (P = 0.02). For all variables, the superficial VOI (1–5 mm) had higher values for BV/TV, Tb.Th, and Tb.N and lower values for Tb.Sp, SMI, Tb.Pf, and DA (P<10^−4^) and the differences between sites were more striking in the first VOI (1–5 mm).

**Fig 2:**
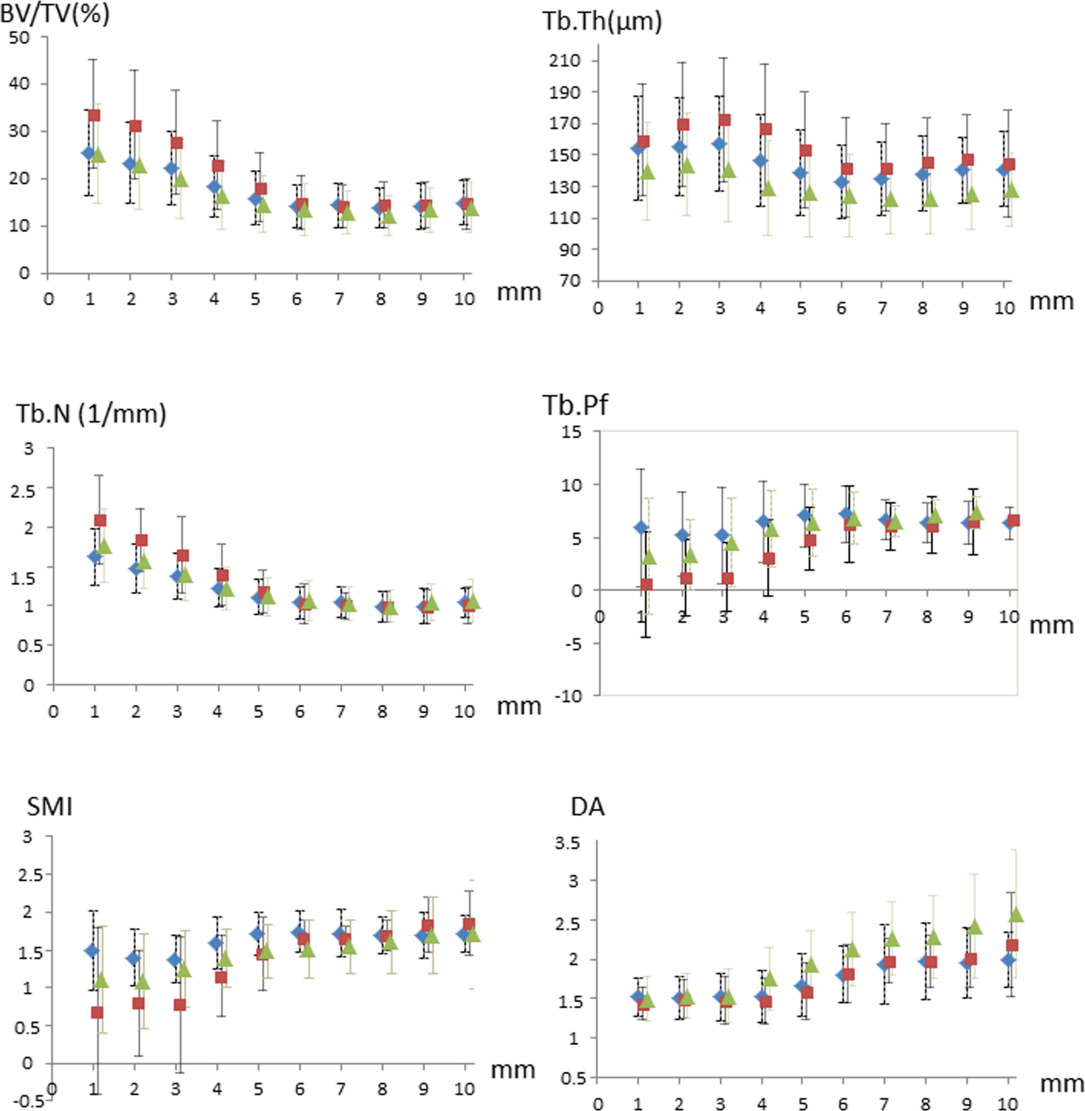
Depth-dependence of morphological variables below the SCP at the three sampling sites. (CENT) central, uncovered; (PER) peripheral, fully covered; (POST) posterior, partially covered. Comparisons were by ANOVA for normally distributed variables and Kruskal-Wallis test otherwise. Data are mean±SD. BV/TV, bone volume fraction (mineralized bone volume/total bone volume); Tb.Th, trabecular thickness; Tb.N, trabecular number; Tb.Pf, trabecular pattern factor; SMI, structure model index; DA, degree of anisotropy, POST, posterior sampling site (partially covered); CENT, central sampling site (uncovered); PER, peripheral sampling site (fully covered).

Fig 2 reports the results of the 1×1mm analysis of variations in variables by depth. For BV/TV, Tb.N, and Tb.Th, a transition zone below which the values no longer changed by depth was identified at 4–6 mm below the SCP (*P*<0.01). The transition zone was at 5–6 mm for DA (*P*<10^−4^) and 3–4 mm for Tb.Pf and SMI (*P*<10^−3^). The depth of the transition zone varied little across the three sites. Deep in the transition zone, the only variables that differed across the three sites were Tb.Th and DA, which agreed with the volumetric analysis (STB_1–5 mm_ vs STB_6–10 mm_).

Table 5 reports the correlations among variables. The only variable significantly correlated with age was Cart.Th at POST (r = -0.40; *P* = 0.03) and CENT sites (r = -0.35; *P* = 0.03) (data not shown). In general, correlations were weak to moderate (r = 0.37–0.64). Cart.Th was correlated with SCP.Por and SCP.Th at only the POST site (ρ = -0.48; P = 0.009 and r = 0.63; P = 0.0003). SCP.Por was correlated with STB microarchitecture variables (BV/TV, Tb.Th, Tb.N, Tb.Pf and SMI) at only the PER site.

**Table 5:** Correlations among SCP and SBT measurements (STB_1–5 mm_) and (STB_6–10mm_).

	Cart.Th POST/CENT/PER	SCP.Th POST/CENT/PER	SCP.Por POST/CENT/PER
SCP.Th	0.63ǂ/ns/ns	-	-
SCP.Por	*-0*.48**/ns/ns	ns/ns/ns	-
**1–5 mm**			
BV/TV	ns/ns/ns	0.37*/*0*.42*/0.62ǂ	ns/ns/-0.51**
Tb.Th	ns/ns/ns	ns/0.64ǂ/0.59ǂ	ns/ns/-0.45*
Tb.N	ns/ns/ns	0.46**/ns/0.50**	ns/ns/-0.38*
Tb.Sp	ns/ns/ns	ns/ns/ns	ns/ns/ns
Tb.Pf	ns/ns/ns	-0.44**/ns/-0.60ǂ	ns/ns/0.50**
SMI	ns/ns/ns	ns/ns/-0.59ǂ	ns/ns/0.50**
DA	ns/ns/ns	-0.39*/ns/-0.39*	ns/ns/ns
**6–10 mm**			
BV/TV	ns/ns/ns	0.37*/ns/0.47**	ns/ns/ns
Tb.Th	ns/ns/ns	ns/0.47**/0.51**	ns/ns/ns
Tb.N	ns/ns/ns	0.48**/ns/ns	ns/ns/ns
Tb.Sp	ns/ns/ns	ns/ns/ns	ns/ns/ns
Tb.Pf	ns/ns/ns	-0.56**/ns/-0.46**	ns/ns/ns
SMI	ns/ns/ns	ns/ns/ns	ns/ns/ns
DA	ns/ns/ns	ns/ns/ns	ns/ns/ns

Pearson correlation coefficients (Spearman correlation coefficients in italics for non-normally distributed data) among trabecular architecture parameters and subchondral plate thickness and porosity at three sampling sites in the medial tibial plateau: central (CENT, uncovered), peripheral (PER, fully covered), and posterior (POST, partially covered)

*P<0.05

**P<0.01

ǂ *P*<10^−3^

ns, nonsignificant

SCP.Th was significantly associated with STB microarchitecture measured at 1–5 mm at the PER site for all variables except Tb.Sp, at the POST site for BV/TV, Tb.N, and Tb.Pf were significantly correlated (r = 0.37–0.62). At the CENT site, SCP.Th was correlated with only BV/TV and Tb.Th. At 6–10 mm, SCP.Th remained correlated with only BV/TV and Tb.Pf at the POST and PER sites, Tb.Th at CENT and PER sites and Tb.N at the POST site.

## Discussion

We simultaneously assessed hyaline cartilage thickness, SCP thickness and porosity, and variables describing STB morphology at the medial tibial plateau of cadaver knees from older individuals with little or no evidence of osteoarthritis. This broad range of measurements provided a comprehensive assessment of the bone–cartilage interface at the medial compartment, which carries 75% of the load across the knee [17] and is the main target of osteoarthritis [19]. Our results show that bone and cartilage structure in the human medial tibial plateau varies considerably across sites that differ in their extent of coverage by the medial meniscus.

At the CENT site, uncovered by the meniscus, the hyaline cartilage and SCP were thicker and the STB architecture was organized to support high loads as compared with the POST and PER sites, which are partially and totally covered by the meniscus, respectively. Within the most superficial 5 mm below the CENT site, BV/TV, Tb.Th, and Tb.N values were higher and Tb.Sp value was lower as compared with the other sites; furthermore, connectivity (as assessed by Tb.Pf) was greater, anisotropy lower, and the trabecular architecture more plate-like in the CENT site. These differences from the PER and POST sites all support greater local bone strength at the CENT site.

We studied cadaver knees with little or no osteoarthritis to obtain a picture of the normal cartilage–bone interface. The most likely explanation for our findings is a protective effect of the meniscus against mechanical loading [17]. The combination of higher values for BV/TV, Tb.Th, and Tb.N and connectivity, with lower values for SMI and Tb.Sp has been described in knees with advanced osteoarthritis [14]. The characteristics of STB in areas uncovered by the meniscus share similarities with those of osteoarthritic STB.

Further evidence for a protective effect of the meniscus can be found in the Outerbridge grades of cartilage degradation in our specimens. At the CENT site, where local loads are greatest [31], the Outerbridge grade was 1 in most knees and 2 in a few knees, whereas at the other two sites, most knees had a grade of 0 and a few a grade of 1. Similar results were obtained from a study of tibial plateaus from seven normal cadaver knees [32].

Most of our results are consistent with those of earlier studies. The mean hyaline cartilage thickness of 2.0±0.42 mm (range 1.28–3.09 mm) in our study was similar to the 2.2-mm value (range 1.7–2.6) obtained in human cadavers by using a needle probe technique [33]. We found the hyaline cartilage over the medial tibial plateau thicker in regions uncovered by the meniscus, as previously reported in both the medial and the lateral plateau [17,32,34,35].

A strength of our study is the detailed analysis of STB architecture using two VOIs located at different depths below the SCP, together with the 1×1mm analysis. Upon visual inspection, two different regions were identified in the subchondral bone, one with more numerous and thicker trabeculae, with a more plate-like structure and horizontal orientation, and the other with a more homogenous microarchitecture. This finding was confirmed by the 1×1mm analysis. In the most superficial layer of about 5 mm, many architectural features differed across the three measurement sites (Fig 2), presumably because of differences in local loads. The values of the morphological and topological parameters changed with depth in the transition zone at each site (PER, POST, and CENT), which indicates changes in mechanical properties. However, the more uniform STB structure at a greater depth probably does not decrease overall mechanical strength but instead disseminates peak forces throughout the trabecular network. The DA results may seem at variance with this interpretation, because the DA value started to increase at a depth of about 4 mm. In the 1×1mm analysis, DA derived from the mean intercept method can be biased by small VOIs, indeed, five trabeculae at a minimum must be included in each direction to obtain reliable measurements [36]. However, regarding the 1–5 and 6–10 mm depths with large VOIs, the DA value was constantly higher at the PER than CENT site and also higher at the 6–10 than 1–5 mm depth.

Architectural anisotropy characterizes the degree of directional organization of a material and is of particular relevance to mechanics–architecture relations [37]. Higher DA values indicate that the orientation more closely parallels the main direction of the compression forces, with few transverse trabeculae parallel to the cartilage surface. This orientation may decrease the ability of the subchondral bone to transmit shear stresses at the PER versus CENT and POST sites and may indicate a decrease in shear stresses from the surface to the depth, consistent with a more uniform trabecular network composed mainly of vertical trabeculae.

Our results are supported by other studies. For instance, in a human autopsy study involving indentation testing, ultimate strength of the proximal tibia abruptly decreased 5 mm below the surface [38]. A study of the equine knees showed greater density of the subchondral bone in regions uncovered than covered by the meniscus [39]. In porcine tibial cartilage, BMD was greater and Tb.Sp lower in uncovered than covered regions [17]. STB data from human autopsy specimens of normal tibias cut 1 mm beneath the SCP were similar to data from our study, with a comparable age subgroup (60–85 years) [14]. Also consistent with our findings are results of a histological study [32] and a 3D micro-CT study [17].

Our study has several limitations. We investigated knees from only older individuals because people who willed their body to science are usually older people; it would be interesting to have data for younger individuals. Delineation of the SCP was open to error, because the calcified zone of the cartilage and cortical endplate were difficult to differentiate. The contrast resolution of the micro-CT images was too low to visualize the tidemark between these two calcified components. SCP porosity was highest at the POST site. Porosity is related to vessel quantity [38], which is higher at joint sites subjected to heavy loads [39]. Porosity provides connections between cartilage and subchondral bone, and high porosity reflects a high level of interactions between these two components. Given that the highest loads at the knee have been reported at the CENT site [40], why porosity was greatest at the POST site in our study is unclear, because this finding seems to contradict the assumption that SCP thickness and vessel density are greatest at the sites of highest loading.

The correlation between SCP.Th and STB microarchitecture was strongest at the PER site, which suggests that the greater cartilage degradation at the CENT site may have disrupted the normal relation between the SCP and STB. Moreover, the relation between Cart.Th and SCP thickness and porosity presented a singular behavior at the POST site as compared with the other sites.

## Conclusions

We observed marked horizontal gradients of cartilage thickness and SCP thickness and porosity in knees from older individuals. Vertical gradients were also observed in the upper 5 mm of the subchondral bone. These gradients probably reflect differences in local loading related in part to the presence or absence of the meniscus. Their existence indicates that cartilage, SCP, and STB should be studied as joint 3D volumes and that the degree of sampling-site coverage by the meniscus should be accurately described. The meniscus probably has a main effect at the most superficial 5 mm below the SCP, because the STB microarchitecture is more uniform at greater depths. The 5-mm depth may be considered a transition zone above which the meniscus, cartilage, SCP, and STB constitute a functional and structural unit. In areas never covered by the meniscus (CENT), the STB microarchitecture was comparable to that in heavily loaded areas, thereby supporting protection by the meniscus against the effects of heavy loading.

## Supporting information

S1 Data data1-5mm. (XLSX)

S2 Data data6-10mm. (XLSX)
